# Effect of Malondialdehyde on the Digestibility of Beef Myofibrillar Protein: Potential Mechanisms from Structure to Modification Site

**DOI:** 10.3390/foods11152176

**Published:** 2022-07-22

**Authors:** Yantao Yin, Lei Zhou, Jiaming Cai, Fan Feng, Lujuan Xing, Wangang Zhang

**Affiliations:** Key Laboratory of Meat Processing and Quality Control, Ministry of Education China, College of Food Science and Technology, Nanjing Agricultural University, Nanjing 210095, China; yantaoyin@126.com (Y.Y.); judgeaie@163.com (L.Z.); jm581117@163.com (J.C.); 2020108066@stu.njau.edu.cn (F.F.); lujuanxing@njau.edu.cn (L.X.)

**Keywords:** protein oxidation, lipid oxidation, malondialdehyde, oxidation sites, in vitro digestion

## Abstract

Lipid oxidation and protein oxidation occur side by side in meat. Here, the effect of malondialdehyde (MDA), the major product of lipid oxidation, on the digestibility of beef myofibrillar proteins (MP) was studied. MP samples were incubated with 0, 0.1, 0.3, 0.5, and 0.7 mM MDA at 4 °C for 12 h and then subjected to in vitro gastrointestinal digestion. The result showed that MDA remarkably reduced the digestibility of MP (*p* < 0.05). MDA treatments significantly increased carbonyl and Schiff base contents in MP (*p* < 0.05). The microstructure observed by atomic force microscopy showed that MDA treatments resulted in the aggregation of MP. Non-reducing and reducing electrophoresis suggested the aggregation was mainly caused by covalent bonds including disulfide bond and carbonyl–amine bond. Proteomics analysis proved that the myosin tail was the main target of MDA attack, meanwhile, lysine residues were the major modification sites. Taken together, the above results imply that MDA induces protein oxidation, aggregation, and blockage of hydrolysis sites, consequently leading to the decrease in both gastric and gastrointestinal digestibility of MP.

## 1. Introduction

Meat, enriched in all essential amino acids, is a good source of high-quality protein for humans [1]. Nevertheless, meat protein is susceptible to oxidation during processing, transportation, and storage [2,3]. The oxidation of meat protein is always triggered by lipid oxidation [4]. Malondialdehyde (MDA) is the main lipid oxidation product in meat and meat products [5]. MDA contains two highly reactive carbonyl groups that can attack nucleophilic amino acid side chains, leading to oxidative damage of proteins [6]. Under physiological conditions, lipid oxidation products including MDA are often involved in oxidative stress, cell metabolism, enzyme activity, and protein–protein interaction [7]. In food systems, MDA-induced oxidative modification has been reported to affect the processing properties and safety of meat. For example, Zhou et al. [8] reported that MDA regulated the gel strength of pork myofibrillar proteins (MP) by forming covalent bonds. Zhu et al. [9] described that MDA-triggered oxidation of chicken sarcoplasmic protein increased advanced glycation end products, which may have a detrimental effect on consumer health.

Digestibility is a critical indicator for evaluating the nutritional value of dietary proteins [10]. On the one hand, the reduction in protein digestibility limits the bioavailability of amino acids. On the other hand, the undigested proteins will be fermented by gut microbiota that produces toxic metabolites, such as phenols, ammonia, and H_2_S, impairing host health [11,12]. Protein oxidation results in modifications of amino acid side chains, which can alter protein structure and even digestibility [2]. The impacts of protein oxidation on digestibility are mainly through two pathways. First, oxidation-induced protein unfolding or aggregation affects the accessibility of digestive enzymes to hydrolysis sites [13]. Second, the oxidation of certain amino acids, such as lysine and arginine, blocks the recognition of digestive enzymes to hydrolysis sites [14]. During the production of air-dried yak meat, Ma et al. [15] reported that protein digestibility decreased along with increases in both lipid and protein oxidation. In a recent study, MDA-induced oxidation was reported to reduce the digestibility of rice bran protein [16].

However, to the best of our knowledge, the influence of MDA-induced oxidation on the digestibility of meat protein has not been well evaluated. Furthermore, protein triggered by MDA oxidation belongs to one of the post-translational modifications, which has gained widespread attention in the field of physiology and medicine [6,7]. Nevertheless, there is little information about the target protein and precise modification sites referring to MDA-MP oxidation. Therefore, the object of this study is to evaluate the influence of MDA on the digestibility of beef MP. To elucidate the mechanism, the effects of MDA on the physicochemical properties and conformation of MP were analyzed, and the MDA-induced exact oxidation sites in MP were identified by mass spectrometry-based proteomics. This work will help to clarify the mechanism of MDA-induced oxidation on beef protein digestibility, providing a theoretical basis for the production of high-quality beef products.

## 2. Materials and Methods

### 2.1. Materials

Semimembranosus from three cattle (250 ± 15 kg) was obtained from Shandong Hongan Co., Ltd. (Binzhou, China) following China’s standards for industrial procedures of slaughter. The reagents of pepsin, trypsin, α-chymotrypsin, 1,1,3,3-tetramethoxypropane (TMP), 2,4-dinitrophenylhydrazine (DNPH), and 8-anilinonapthalene-1-sulfonic acid (ANS) were purchased from Sigma-Aldrich Co., Ltd. (Shanghai, China).

### 2.2. MDA Solution

Fresh MDA solution was prepared as the method described by Wu et al. [17]. Briefly, 16.8 mL of TMP was hydrolyzed with 20 mL of 5 M HCl in 63.2 mL of ultrapure water. Then, the mixture was incubated under darkness at 40 °C for 40 min. The pH of the hydrolysate was then adjusted to 6.0 using 6 M NaOH. The absorbance was determined using a UV spectrophotometer (Hitachi, Tokyo, Japan) at 267 nm. The molar absorption coefficient of 31,500 mol^−1^ cm^−1^ was used for calculating the concentration of MDA solution.

### 2.3. Preparation of MP

The extraction of MP was accorded to a previous study [18]. Beef semimembranosus (40 g) was homogenized at 12,000 rpm for 2 × 30 s with 200 mL of extraction buffer I (1 mM EGTA, 2 mM MgCl_2_, 100 mM NaCl, and 10 mM potassium phosphate, pH 7.0). The buffer I was first pre-cooled in a refrigerator at 4 °C for 12 h, and buffer I was under ice–water during the homogenate process. Then, the homogenate was centrifuged (Avanti J-25i, Beckman Coulter, CA, USA) at 2000× *g* (4 °C, 15 min). The above operations were repeated three times using buffer I. The pellet was further homogenized (12,000 rpm, 2 × 30 s) with 200 mL of extraction buffer II (0.1 M NaCl, pH 6.0) under ice–water. The homogenate was filtered through two layers of gauze followed by centrifugation (4 °C, 2000× *g*, 15 min). The pellet was washed three times with buffer II to obtain MP. The obtained MP was solubilized in buffer III (0.6 M NaCl, 20 mM Na_2_HPO_4_/NaH_2_PO_4_, pH 6.0), and treated with MDA immediately. The MP concentration was measured using a BCA protein assay kit (Thermo Scientific, Rockford, IL, USA).

### 2.4. Incubation of MP with MDA

The MP suspension (10 mg/mL) was mixed with 0 (CK), 0.1, 0.3, 0.5, and 0.7 mM MDA. After vortexing, the mixtures were incubated under darkness for 12 h at 4 °C. Then, each sample was dialyzed at 4 °C for 12 h using a 3-kDa dialysis bag to remove the excess MDA. The dialyzed solution was then freeze-dried and stored at −80 °C for further analysis.

### 2.5. Carbonyl Content

Carbonyl content was determined by DNPH following the method described by Zhang et al. [19]. Briefly, 4 mL of sample suspension (5 mg/mL) was mixed with 4 mL of 10 mM DNPH solution (dissolved in 2 M HCl), and then incubated at 25 °C for 1 h. Then, the mixture was precipitated with 5 mL of trichloroacetic acid followed by centrifugation for 10 min at 12,000× *g* at 4 °C. The precipitate was washed 5 times with 4 mL of ethanol and ethyl acetate (1:1, *v*/*v*). Then, the pellet was dissolved in 2 mL of 6 M guanidine solution and centrifuged for 10 min at 8000× *g*. The sample incubated with 2 M HCl instead of DNPH solution served as blank. The carbonyl content was calculated with the absorption coefficient (22,000 M^−1^ cm^−1^).

### 2.6. Schiff Base

The content of Schiff base was evaluated by fluorometry [20]. Three milliliters of sample (1 mg/mL) were mixed with 3 mL of 16 M urea (dissolved in 20 mM PBS). After the vortexing, the emission spectrum was recorded by a microplate reader (M2, Molecular Devices, CA). The excitation wavelength was set as 350 nm and the emission wavelength was 460 nm.

### 2.7. Secondary Structures

The secondary structures of MP were determined by circular dichroism (CD) spectroscopy (J-1500, Jasco Corporation, Tokyo, Japan). Sample solutions (0.2 mg/mL) were scanned in 190–260 nm at a scan rate of 100 nm/min. The proportions of secondary structures (%) were calculated using CD Pro software.

### 2.8. Intrinsic Tryptophan Intensity

The tryptophan fluorescence of MP was measured using an M2 microplate reader. The emission spectra of tryptophan were recorded from 300 to 400 nm at 283 nm excitation wavelengths with sample solutions (0.5 mg/mL) [21].

### 2.9. Surface Hydrophobicity (S_0_)

The S_0_ was measured according to the method described by Chen et al. [22]. First, 4 mL of sample (1 mg/mL) was mixed with 20 μL of 15 mM ANS (20 mM PBS, pH 7.0). Then, the mixture was incubated at 25 °C for 25 min under darkness. The fluorescence intensity was recorded using an M2 microplate reader at the excitation wavelength of 380 nm and emission wavelengths from 410 to 570 nm.

### 2.10. Sodium Dodecyl Sulfate-Polyacrylamide Gel Electrophoresis (SDS–PAGE)

The sample (2 mg/mL) was mixed with an equal volume of loading buffer (20% glycerol, 0.125 M Tris, 4% SDS, pH 6.8). The reducing SDS–PAGE was with 5% β-mercaptoethanol in the loading buffer, while non-reducing without β-mercaptoethanol in the loading buffer. The mixture was boiled at 95 °C for 5 min. Then, 8 μL of sample or 6 μL of marker protein (Thermo Fisher) was loaded into each lane of a 4–10% gradient gel. The electrophoresis was conducted by a Bio-Rad Mini-Protean Tetra System (Bio-Rad Laboratories, Hercules, CA) running at 70 V for 30 min followed by 110 V for 70 min. Then, the gel was stained with Coomassie brilliant blue for 30 min and decolorized with decolorizing solution (10% acetic acid, 10% methanol; 1:1) for 12 h.

### 2.11. Atomic Force Microscopy (AFM)

The morphology of the sample was observed using AFM (Bruker, Karlsruhe, Germany) as the method described by Xu et al. [23]. Briefly, 5 μL of the sample (0.05 mg/mL) was coated onto freshly cleaved mica and was dried under ambient temperature. Then, the image was acquired by AFM with tapping mode, and the scan frequency was 1.0 Hz.

### 2.12. In vitro Digestion and Protein Digestibility

The in vitro simulated gastric fluid (SGF) and the simulated intestinal fluid (SIF) were prepared according to the standard described by Minekus et al. [24]. For gastric digestion, a 20 mg freeze-dried sample was dissolved in 4 mL of SGF. Then, pepsin was added to the mixture to achieve an enzymatic activity of 2000 U/mL to start gastric digestion. The mixture was shaken in a shaker under 37 °C at 150 rpm. After gastric digestion for 120 min, 1 mL of digested chyme was collected and immediately mixed with the same volume of SIF to inactivate pepsin. For intestinal digestion, 3 mL of digested chyme was mixed with 3 mL of SIF, and then α-chymosin and trypsin were added to achieve final enzymatic activities of 25 U/mL and 100 U/mL, respectively. The mixture was also reacted in a shaker under 37 °C at 150 rpm for 2 h. Then, the gastrointestinal digest was heated at 95 °C (5 min) to inactivate α-chymosin and trypsin.

Protein digestibility was determined by degree of hydrolysis (DH) following our previous work [25]. Briefly, 75 μL of the digestion was precipitated with an equal volume of TCA (24%) in ice water for 30 min. The mixture was then centrifuged (4000× *g*, 20 min, 4 °C). Subsequently, 30 μL supernatant/standard L-leucine (0.05–3 mM) was mixed with 900 μL sodium tetraborate (0.1 M, pH 8.0) and 300 μL fluorescamine acetone solution (0.2 mg/mL). Fluorescence was measured using a microplate reader (M2, Molecular Devices Ltd., CA, USA) at an excitation wavelength of 390 nm and an emission wavelength of 480 nm. The DH was calculated as follows:

$$\mathrm{DH}=\frac{\;[-{\mathrm{NH}}_{2}\;(t)]\;-\;[-{\mathrm{NH}}_{2}\;(0)]}{[-{\mathrm{NH}}_{2}\;(\infty )]\;-\;[-{\mathrm{NH}}_{2}\;(0)]}\;\times \;100\%$$

[-NH_2_ (t)] means the concentration of primary amines after gastric/gastrointestinal digestion. [-NH_2_ (0)] means the concentration of primary amines before digestion. [-NH_2_ (∞)] was measured from the fluorescence of each sample (before digestion) fully hydrolyzed with 6 M HCl at 100 °C for 48 h.

### 2.13. Identification of Modification Sites

A proteomics method was performed to identify the modification sites between MP and MDA. First, 10 μL of sample (5 mg/mL) was mixed with 15 μL of 100 mM DTT and 60 μL of ammonium bicarbonate solution (50 mM). Then, the mixture was boiled at 95 °C for 5 min. Then, 12 μL of 100 mM iodoacetamide was added to the mixture and incubated in a dark environment (20 min). Subsequently, trypsin (0.1 mg/mL) was used to hydrolyze the protein at 37 °C for 18 h. The Surveyor LC system (Thermo Finnigan, San Jose, CA, USA) combined with an RP-C18 sampling column (Column technology Inc., Fremont, CA, USA) and a Zorbax 300SB-C18 peptide trap separation column (Agilent Technologies, Wilmington, DE, USA) was used for the fractionation of the peptide mixture. The eluents used were 0.1% formic acid aqueous solution (eluent A, *v*/*v*) and 84% acetonitrile aqueous solution containing 0.1% formic acid (eluent B, *v*/*v*). The gradient elution parameters were: a linear increase from 4% to 50% and 50% to 100% and maintenance at 100% eluent B from 0–50 min, 50–54 min, and 54–60 min, respectively. Peptides were identified using a Q Exactive mass spectrometer (Thermo Fisher) under positive ionization mode. The obtained tandem mass spectra data were searched using MaxQuant software (version 1.5.5.1) with a false discovery rate of 1%. The fixed modification was carbamidomethyl and the variable modifications were C_3_H_2_O (+54 Da) and C_8_H_6_O_2_ (+134 Da) based on previous studies [26,27]. The peptide tolerances and MS/MS were set as 20 ppm and 0.1 Da, respectively.

### 2.14. Statistical Analysis

Statistical differences between groups were evaluated by one-way analysis of variance followed by Duncan’s post-test with SPSS 23.0 (SPSS Inc., Chicago, IL, USA). The significance level was noted when *p* < 0.05. Results were presented as mean ± standard deviation. Protein accession number was collected from http://www.uniprot.org (accessed on 10 June 2021) (UniProt database) and visualized in a heat map using the *Heml* 1.0 software (hemi.biocuckoo.org (accessed on 17 July 2021).

## 3. Results and Discussion

### 3.1. Protein Oxidation Level

#### 3.1.1. Carbonyl and Schiff Base

**Figure 1:**
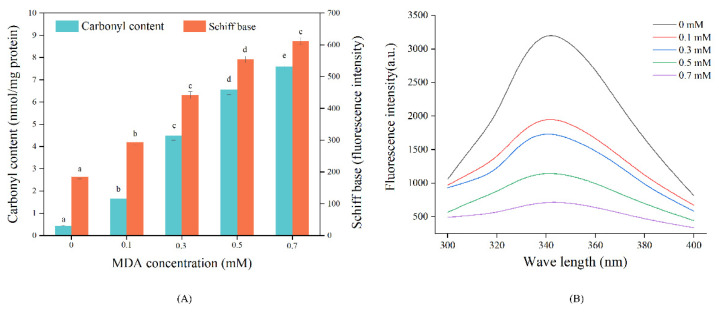
Effect of MDA on MP carbonyl, Schiff base content (**A**), and tryptophan endogenous fluorescence (**B**). Different letters (a, b, c, d, e) indicate a significant difference (*p* < 0.05, *n* = 5).

The carbonyl content and Schiff base products are two important indicators to reflect protein oxidation level [28]. As shown in Figure 1A, both the carbonyl and Schiff base contents were significantly increased with increasing MDA concentration. These results indicated that MDA significantly increased the oxidation of MP. The study of Wang et al. [29] also found that MDA treatment elevated carbonyl content and promoted the formation of Schiff base products in soy protein isolates. The increased carbonyl content could be attributed to the introduction of the carbonyl group from MDA by covalent binding [4]. MDA could react with nucleophilic amino acid side chains, such as lysine, and arginine, due to the presence of two active aldehyde groups [8]. Schiff base products are generated from the reactions between carbonyl and amino groups [30]. It has been demonstrated that the aldehyde group in MDA can react with the ε-amino group of proteins, forming Schiff base adducts [31]. Therefore, the covalent reactions between nucleophilic amino acid side chains and MDA might be the primary reason for the oxidation of MP [28].

#### 3.1.2. Intrinsic Tryptophan Intensity

Tryptophan is highly sensitive to oxidation and its intrinsic fluorescence is also commonly used to assess protein oxidation [21]. As shown in Figure 1B, MDA treatments resulted in a sharp decrease in MP tryptophan fluorescence intensity, producing a 39.04% fluorescence loss in the 0.1 mM MDA treated sample. Moreover, almost no fluorescence was observed in the sample treated with 0.7 mM MDA. A similar phenomenon was observed in MDA-treated rabbit MP [4]. The decline in fluorescence intensity might be due to the oxidative loss of tryptophan [21]. It has proved that the indole nitrogen of tryptophan is vulnerable to MDA attack [32]. Additionally, MDA-induced conformational changes could also lead to the reduction in tryptophan fluorescence of MP [16].

### 3.2. Secondary Structures

**Figure 2:**
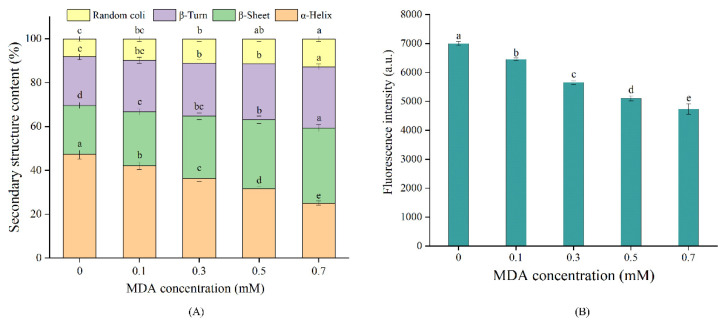
Effect of MDA on MP secondary structures (**A**), surface hydrophobicity (**B**). Different letters (a, b, c, d, e) indicate a significant difference (*p* < 0.05, *n* = 5).

The influence of MDA treatments on MP secondary structures is shown in Figure 2A. The CK sample contained 47.24%, 22.19%, 22.41%, and 8.16% of α-helix, β-turn, β-sheet, and random coil structures, respectively. The proportion of α-helix decreased from 47.24% to 25.07% with MDA concentration increasing from 0 to 0.7 mM, while the proportion of β-sheet increased from 22.41% to 34.29%. These changes in secondary structures might be associated with the modifications of amino acid side chains mediated by MDA. It is generally accepted that the α-helix structure is stabilized by hydrogen bonds between NH- and -CO [33]. Li et al. pointed out that the covalent attachments of MDA with free amino groups would decrease the number of hydrogen bonds and thus destroy the α-helix structure [34]. MDA-induced oxidation is one of the non-enzymatic post-translational modifications (PTM) [6]. Such modification may result in changes in charge content or distribution, and appearance/blockage of key interaction residues of the protein, thus altering the structure of proteins [35]. In the present study, MDA-induced unfolding of α helix structure exposed hydrophobic amino acids that were originally buried in MP, which might alter inter/intramolecular interactions and thus contribute to the increase in β-turns and β-sheets structures [30]. In addition, the β-sheet structure is related to the self-aggregation of proteins [36]. MDA-induced PTM in lysine residue might disrupt the original hydrophilic/hydrophobic balance of MP which in turn increased the intrinsic aggregation, thereby facilitating the formation of β-sheet [7,35]. Our results agree with the study of Song et al. [30], who reported MDA treatments reduced the α-helix proportion and increased the β-sheet proportion in the tropomyosin of shrimp.

### 3.3. Surface Hydrophobicity

Hydrophobic amino acids are generally buried in the interior of proteins, and S_0_ is usually used for characterizing protein conformation [23]. As shown in Figure 2B, S_0_ gradually decreased with the increase in MDA concentration (*p* < 0.05), reaching a 32.29% decrease in the 0.7 mM MDA treated sample. The changes in S_0_ are correlated with the balance between protein unfolding and aggregation. The decrease in S_0_ in this work suggested that MDA induced aggregation of MP, which reduced the accessibility of ANS to hydrophobic amino acids, (e.g., tyrosine and phenylalanine). Supporting this view, Wu et al. [17] reported that MDA-induced soybean protein aggregation declined the value of S_0_. Moreover, the covalent binding with MDA introduced hydrophilic groups, (e.g., carbonyl group) to MP, which might break the original hydrophilic/hydrophobic balance, thus reducing S_0_. Our results are in line with the study of Li et al. [16], who reported the S_0_ of rice bran protein was significantly decreased with MDA treatment. Conversely, an increase in S_0_ was observed in whey protein isolate [37], and shrimp tropomyosin [30], after MDA treatment. This discrepancy is probably related to the variances in protein structure and degree of modification [6].

### 3.4. SDS-PAGE

**Figure 3:**
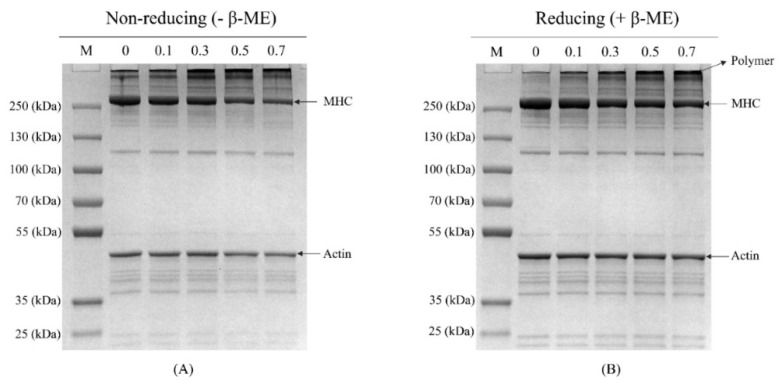
Effect of MDA on MP proteins profile. Non-reducing condition (**A**), reducing condition (**B**). M, protein marker; MHC, myosin heavy chain.

Non-reducing and reducing electrophoreses were conducted to visualize the changes in protein profile. All samples exhibited the typical electrophoresis profile of myofibrillar protein, with myosin heavy chain (MHC) and actin as the dominant polypeptides. Under non-reducing conditions, the band intensity of MHC gradually decreased with increasing MDA concentration, especially for 0.5 and 0.7 mM MDA treated samples (Figure 3A). Simultaneously, there was a gradual increase in polymers on the top of the stacking gel. This phenomenon could be due to the formation of large aggregates that could not migrate into the stacking gel [8]. Under reducing conditions (Figure 3B), the lost MHC bands were recovered in the CK sample, suggesting the polymer was mainly formed by disulfide bonds [38]. It was noteworthy that, regarding MDA treated samples, there were still obvious polymers on the top of stacking gel. These results suggested that, in addition to the disulfide bond, other covalent bonds also participated in the formation of aggregate. This phenomenon could attribute to the formation of carbonyl–amine covalent bonds with reactions between MDA and nucleophilic amino acid side chain [8]. The dose-dependent increase in carbonyl content and Schiff base products (Figure 1A) in the MDA-treated MP samples supported this speculation.

### 3.5. Microstructure

**Figure 4:**
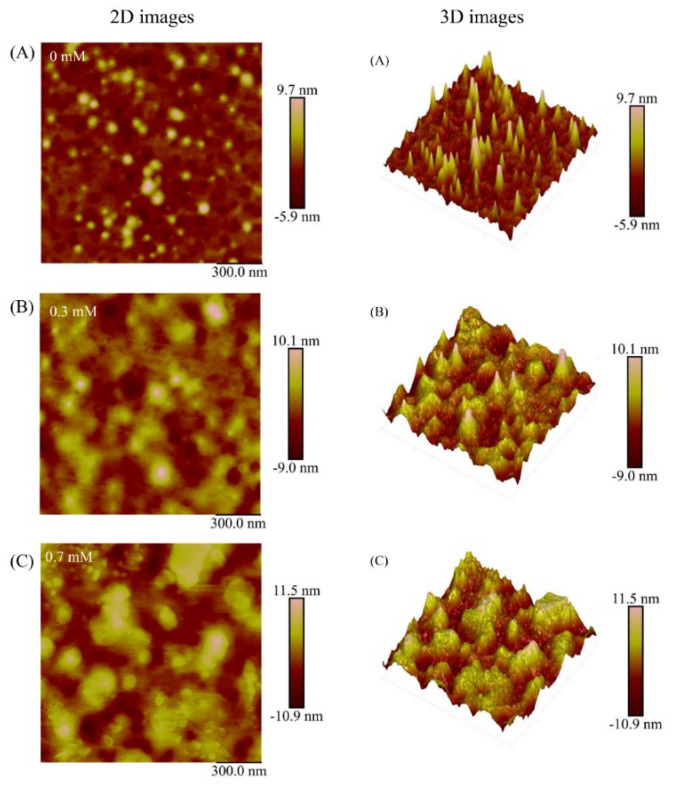
Effect of MDA on the morphology of MP. The 0 mM MDA treated sample (**A**), 0.3 mM MDA treated sample (**B**), 0.7 mM MDA treated sample (**C**). The scan size is 1.5 μm × 1.5 μm.

AFM was performed to observe the effect of MDA on the microstructure of MP. The morphologies of 0, 0.3, and 0.7 mM MDA treated samples are presented in Figure 4. The CK sample exhibited small size, low height, and uniformly dispersed spherical particles. After modification with MDA, the samples presented aggregation characterized by larger particle size, greater height, and broadly distributed particle clusters. For the 3D display, compared with the CK sample, the 0.3 mM MDA treated sample presented a larger mountain-like appearance, and this phenomenon was further exacerbated in the 0.7 mM MDA treated sample. These changes are consistent with the results of SDS-PAGE (Figure 3), providing strong evidence that MDA led to the aggregation of MP. As mentioned above, such aggregation could be attributed to the formation of covalent bonds, such as disulfide bonds and carbonyl–amine. Additionally, the electrostatic interactions might also participate in the aggregation. Petrov and Zagrovic [39] described that the formation of carbonylation at basic amino acid residues (lysine, arginine) would result in the removal of positive charge at these sites, thus contributing to protein aggregation. In addition, the increased β-sheet structure provided a favorable environment to enhance intra- and intermolecular interactions, which further facilitated the progress of aggregation [7].

### 3.6. Protein Digestibility

**Figure 5:**
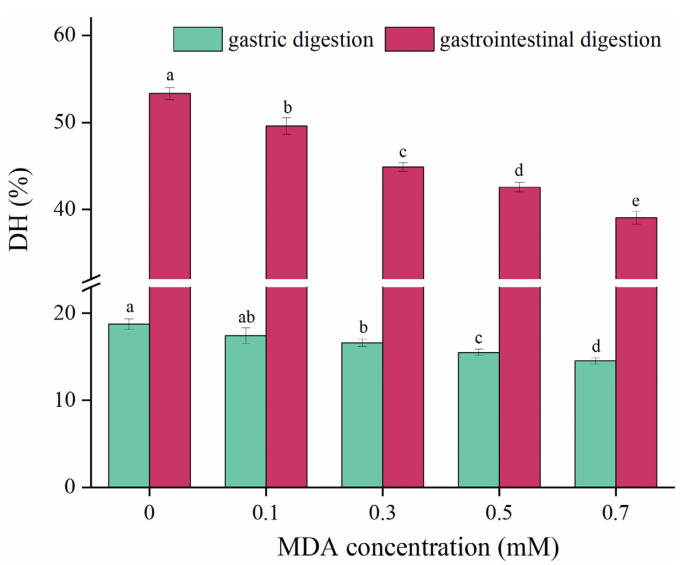
Effect of MDA on MP gastric and gastrointestinal digestibility. Different letters (a, b, c, d, e) indicate a significant difference (*p* < 0.05, *n* = 5).

In vitro simulated gastrointestinal digestion is a convenient way to study the digestive properties of foods, and DH is widely used to characterize protein digestibility [24,25]. For the CK sample, the DH was 18.75% and 53.32% in gastric and gastrointestinal digestion, respectively (Figure 5). With increasing MDA concentration, DH markedly decreased in both gastric and gastrointestinal digestion. Compared with the CK sample, the DH decreased by 26.78% in the 0.7 mM MDA treated sample at gastrointestinal digestion. These results indicated that MDA remarkably reduced the digestibility of MP. Our results agree with previous studies, which reported MDA reduced the digestibility of soy protein isolate [40], whey protein isolate [35], and rice bran protein [16]. This phenomenon can be explained from two aspects. First, protein aggregation buried hydrolysis sites and contributed to the reduction of MP digestibility. Second, Luna and Estévez [14] pointed out that protein carbonylation could block the recognition of active hydrolysis sites by digestive enzymes. Tryptophan residues are one of the preferred cleavage sites for pepsin, and trypsin is preferred to cleave at residues of lysine and arginine [41]. Thus, MDA-induced PTM at *amino* *acid* sites of tryptophan, lysine, and arginine could also be responsible for the decline of MP digestibility [35].

### 3.7. Modification Sites

**Figure 6:**
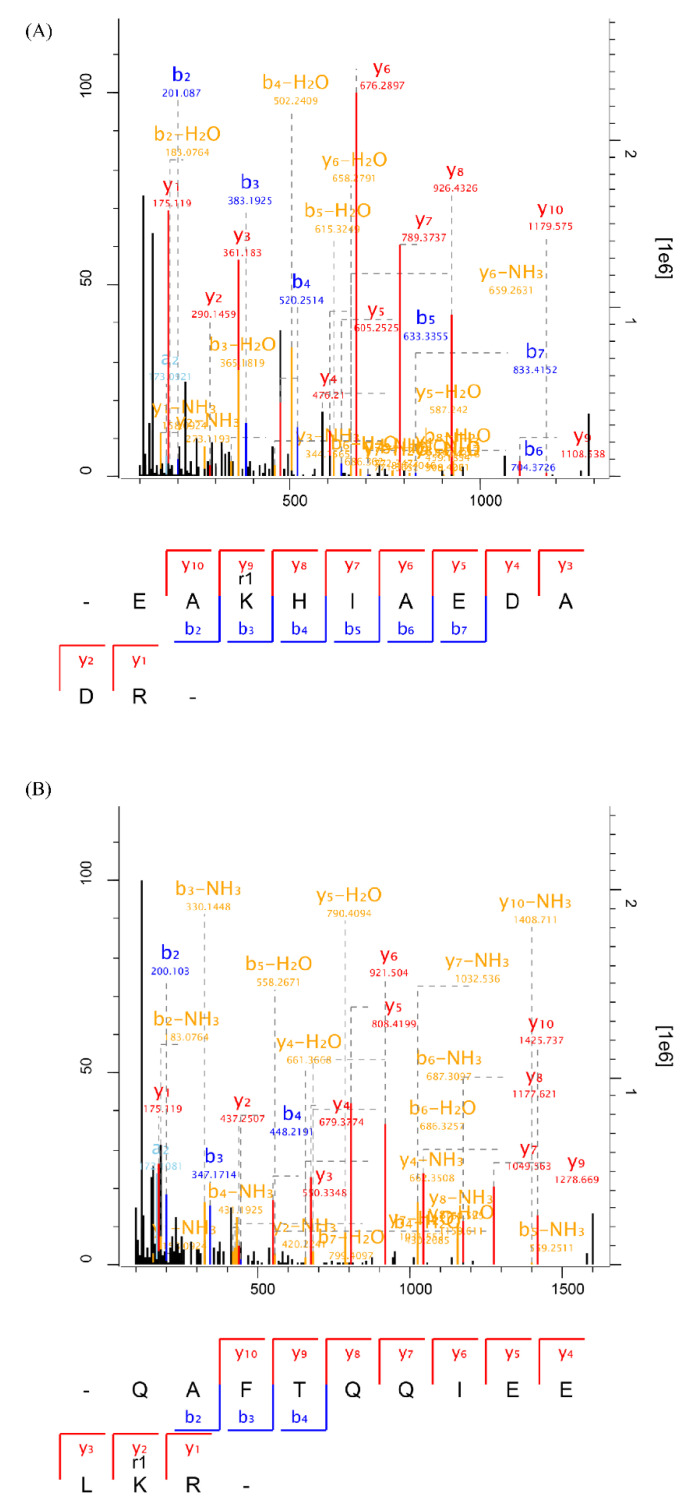
Representative MS/MS spectrums of MDA-modified peptides. (**A**) The peptide modified with the formation of Schiff base adduct at lysine (K); (**B**) the peptide modified with the formation of DHP adduct at lysine (K).

MDA-induced modification sites in MP were identified to better explore the underlying molecular mechanism of decreased digestibility. The representative mass spectrums of modified peptides at lysine are presented in Figure 6. The modified peptide with an increase of 54 Da (Figure 6A) and 134 Da (Figure 6B) indicated the formation of Schiff base and dihydropyridine (DHP)-type adducts, respectively [27]. The number of identified modification sites is shown in Figure S1A. There were 51 modification sites (36 Schiff base-type adducts, 15 DHP-type adducts) identified in the CK sample, which might be attributed to the generation of endogenous MDA produced in postmortem [28]. Compared with the CK sample, the number of modification sites remarkably increased after MDA treatment, with 186 and 284 modification sites identified in 0.3, 0.7 mM MDA treated samples (Figure S1A). These results correspond well to the increase in Schiff base products (Figure 1A). Additionally, it was noted that the modification sites mainly occurred at lysine residues, which accounted for 82.80%, 91.90% of the total modification sites in 0.3, 0.7 mM MDA treated samples (Figure S1B). This result agrees with a previous study, reporting that the ε-amino group of lysine was the main target when exposed human serum albumin to MDA [27]. It has been confirmed that the Maillard reaction on lysine residues can block the cleavage site for trypsin [42]. Likewise, the covalent binding between MDA and lysine might also cover the cleavage sites for trypsin, thus resulting in the reduction in MP digestibility. Additionally, the formation of Schiff base and DHP adducts also increased steric hindrances of cleavage sites for digestive enzymes [41]. Furthermore, both pepsin and trypsin have been reported to have secondary enzyme–substrate interactions [43,44]. Thus, the formation of adducts at the nearby cleavage sites, (e.g., asparagine, glutamine; Figure S1B) might also contribute to the decline in digestibility by reducing protease hydrolysis kinetics.

**Figure 7:**
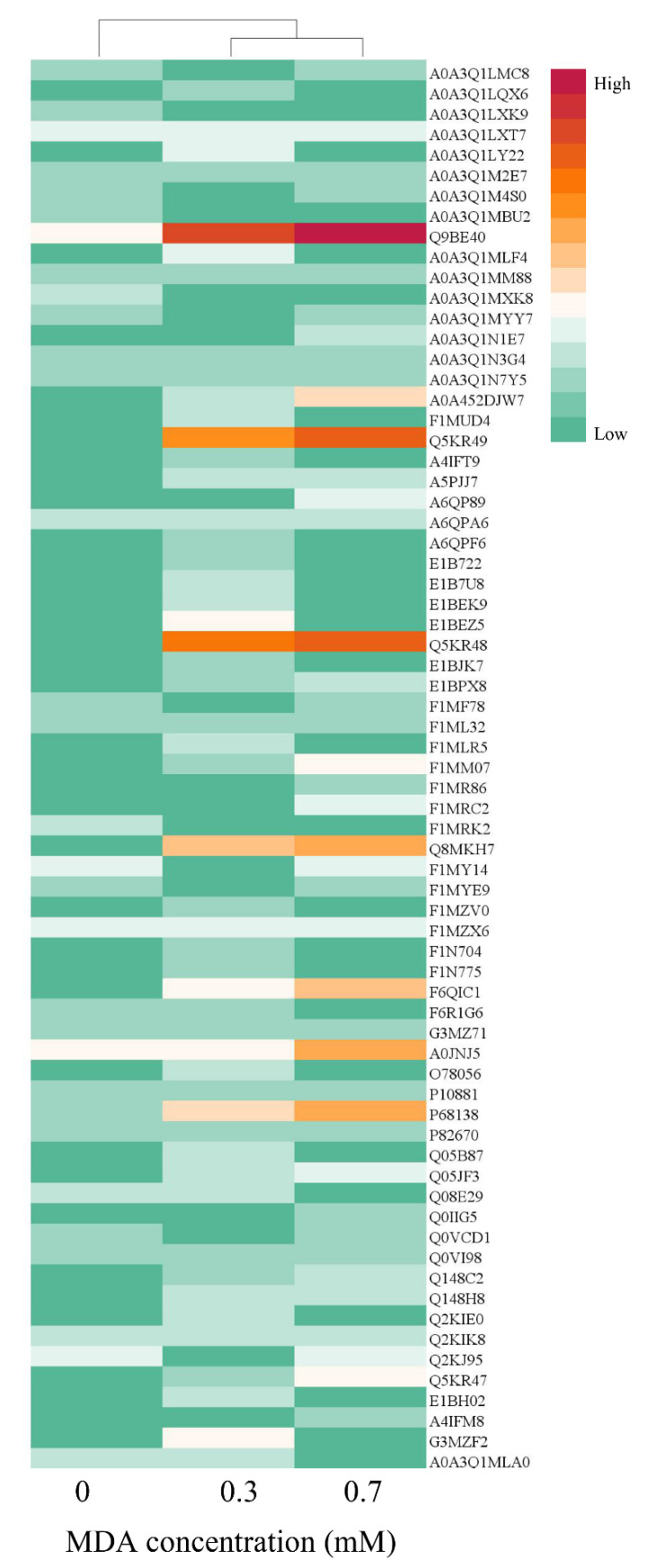
The MDA modified protein profiles displayed by heat map. The red color indicates more modification sites in the corresponding protein, followed by orange, and the green color indicates fewer modification sites.

**Figure 8:**
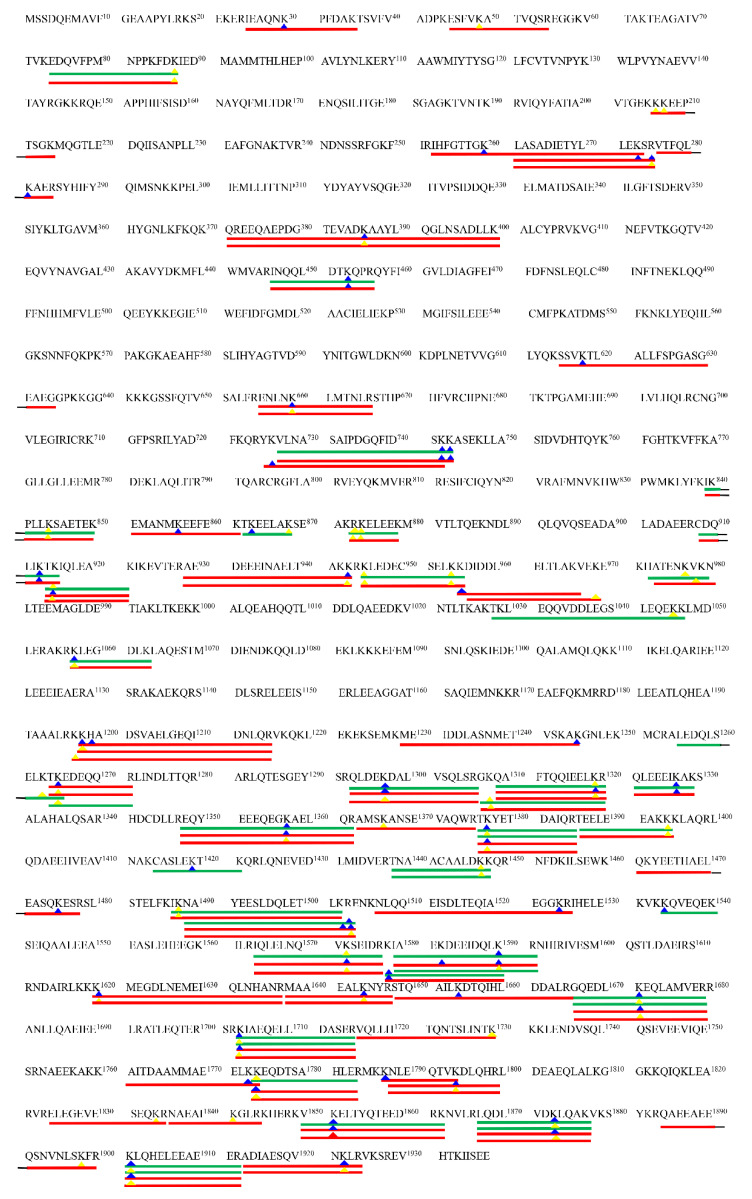
Distributions of the modification sites in myosin-1. The green and red lines indicate the identification of modified peptides in 0.3 mM and 0.7 mM MDA treated samples, respectively. The blue and yellow triangles indicate the binding sites formed with Schiff base and DHP adducts, respectively. The amino acids from the 1st to 844th constitute the myosin-1 head region; the amino acids from the 845th to 1938th constitute the myosin-1 tail region.

The modified protein profile was analyzed to explore the target of MDA-oxidized protein. As shown in Figure 7, the red color represents more modification sites identified in this protein, followed by orange and green. The red and orange colors were mainly presented in 0.3, 0.7 mM MDA treated samples, and they were clustered into one group, separating from the CK sample. The proteins of Q9BE40 (myosin-1), Q5KR49 (tropomyosin alpha-1 chain), Q5KR48 (tropomyosin beta chain), Q8MKH7 (troponin T), F6QIC1 (troponin I2), A0JNJ5 (myosin light chain 1/3), and P68138 (actin) exhibited notable different colors among the three groups (Figure 7). Myosin was the most vulnerable protein to MDA attack among these modified proteins. In the CK sample, there were 11 modification sites identified in myosin from the CK sample, while the number increased to 60 in the 0.3 mM MDA treated sample. For the 0.7 mM MDA treated sample, the number of modification sites rose to 135, which increased 11.27-fold compared with the CK sample. The different susceptibility of proteins to MDA modification might attribute to their discrepancy in molecular properties, including amino acid composition, structure, charge, and protein location [6,45]. For example, myosin exhibits a coiled structure, while actin exhibits a globular one. Similarly, during meat cooking, myosin was more vulnerable to oxidative modification compared to other proteins in pork myofibrillar proteins [46]. Given that myosin-1 was the major modified protein, the precise localization of modification sites in myosin-1 was mapped to obtain further information about modification regions (Figure 8). Interestingly, it was found that more modification sites were identified in the tail region of myosin-1 than in the head region. For example, in the 0.3 mM MDA treated sample, there were 38 modification sites identified in the myosin-1 tail, whereas there were only 5 in the head (Figure 8). This result indicates the myosin-1 tail is more vulnerable to MDA attack than the head region. This phenomenon could attribute to the differences in amino acid composition of myosin-1 head and tail [6]. According to the hydrophobicity/hydrophilicity analysis by the ExPASy ProtScale database, the hydrophilicity of the myosin-1 tail is much stronger than that of the head zone (Figure S2). The higher hydrophilicity in the myosin-1 tail could provide a more favorable environment for the diffusion of MDA and thus increase the efficiency of modifications. Our previous study showed that the released peptides were mainly originated from myosin during the gastrointestinal digestion of beef [25]. Moreover, Zhao et al. [47] pointed out that most (78%) of the released myosin peptides originated from the tail region. Thus, MDA-induced oxidative modification of myosin, especially in the tail region, could be the primary reason for the decrease in MP digestibility.

## 4. Conclusions

The present study reveals MDA significantly reduces both gastric and gastrointestinal digestibility of MP. Upon MDA treatment, the carbonyl, Schiff base content, and β-sheet structure significantly increased, while the tryptophan endogenous fluorescence and α-helix structure decreased in MP. These changes in physicochemical properties, accompanied by the formation of covalent bonds (disulfide bond and carbonyl–amine bond), resulted in the aggregation of MP. Proteomic analysis verified the covalent binding between MDA and MP. The lysine residue was the main binding site and myosin (especially the tail region) was the main target protein of modification. Therefore, MDA-induced oxidative modification caused aggregation and blockage of active hydrolysis sites, which increased the proteolysis resistance of MP, eventually leading to the decline in digestibility. The control strategies, such as adding phytochemicals, to improve the MDA-mediated reduction in meat protein digestibility are required.

## Data Availability

Data is contained within the article or supplementary material.

## Supplementary Materials

The following are available online at https://www.mdpi.com/article/10.3390/foods11152176/s1, Figure S1: The identification of MDA-induced modification sites in MP using a proteomics method. (A) the total number of modification sites; (B) the distribution of the modification sites separately on histidine (H), asparagine (N), glutamine (Q), arginine (R), and lysine (K); Figure S2: The hydrophobic indexes of myosin-1 predicted with the ExPASy ProtScale database. The positive values indicate hydrophobic regions and negative values indicate hydrophilic regions. For convenience display, 1938 amino acids in myosin-1 were divided into groups with each of 50 amino acids (i.e., group 1 = amino acids 1−50, group 2 = amino acids 51−100, …, group 39 = amino acids 1900−1938). The 1st to 16.88th groups corresponded myosin-1 head region; the 16.89th to 39th groups corresponded myosin-1 tail region.
