# Supplementary material for: Effect of Malondialdehyde on the Digestibility of Beef Myofibrillar Protein: Potential Mechanisms from Structure to Modification Site
Source: Foods. 2022 Jul 22;11(15):2176. doi: 10.3390/foods11152176 (PMC9330876; doi:10.3390/foods11152176)
Supplement: Supplementary file 1 [file foods-11-02176-s001.zip › foods-1814691-supplementary.pdf]

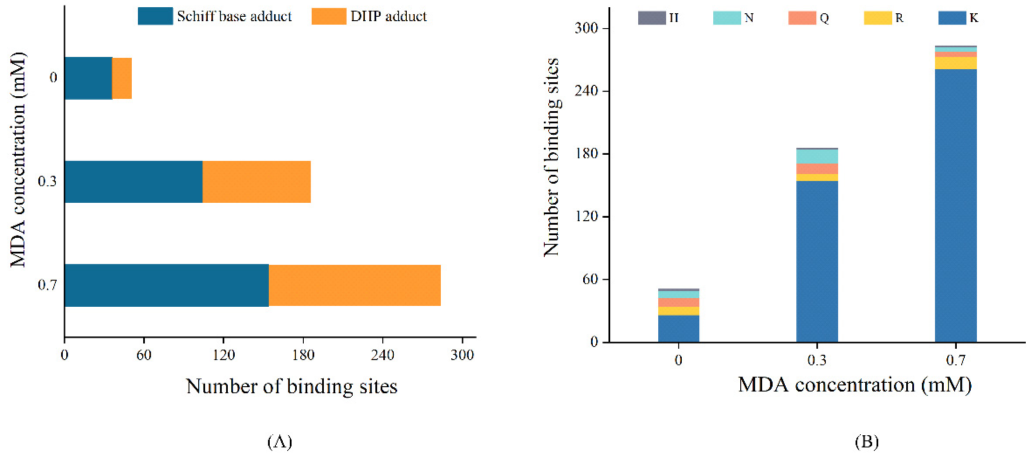

Figure S1. The identification of MDA-induced modification sites in MP using a proteomics method. (A) the total number of modification sites; (B) the distribution of the modification sites separately on histidine (H), asparagine (N), glutamine (Q), arginine (R), and lysine (K).

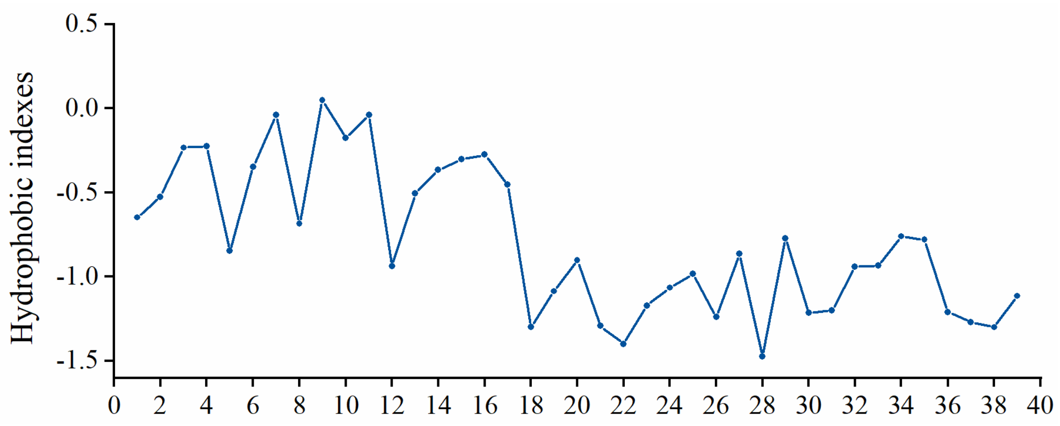

Figure S2. The hydrophobic indexes of myosin-1 predicted with the ExPASy ProtScale database. The positive values indicate hydrophobic regions and negative values indicate hydrophilic regions. For convenience display, 1938 amino acids in myosin-1 were divided into groups with each of 50 amino acids (i.e., group 1 = amino acids 1−50, group 2 = amino acids 51−100, ..., group 39 = amino acids 1900−1938). The 1st to 16.88th groups corresponded myosin-1 head region; the 16.89th to 39th groups corresponded myosin-1 tail region.
